# The characteristics of brain atrophy prior to the onset of Alzheimer’s disease: a longitudinal study

**DOI:** 10.3389/fnagi.2024.1344920

**Published:** 2024-05-28

**Authors:** Ying Hu, Ting Zhu, Wei Zhang

**Affiliations:** ^1^Department of Radiology, West China Hospital, Sichuan University, Chengdu, China; ^2^West China Biomedical Big Data Center, West China Hospital, Sichuan University, Chengdu, China; ^3^Mental Health Center of West China Hospital, Sichuan University, Chengdu, China; ^4^Med-X Center for Informatics, Sichuan University, Chengdu, China

**Keywords:** Alzheimer’s disease, brain atrophy, dementia, 3D T1, longitudinal study

## Abstract

**Objective:**

We aimed to use the onset time of Alzheimer’s disease (AD) as the reference time to longitudinally investigate the atrophic characteristics of brain structures prior to the onset of AD.

**Materials and methods:**

A total of 328 participants from the ADNI database with clear onset of AD and structural imaging data were included in our study. The time before the onset of AD (abbreviated as BAD) was calculated. We investigated the longitudinal brain changes in 97 regions using multivariate linear mixed effects regression models.

**Results:**

The average BAD was −28.15 months, with a range from −156 to 0 months. The 54 brain regions showed significant atrophy prior to the onset of AD, and these regions were mainly distributed in the frontal and temporal lobes. The parietal and occipital lobe exhibited relatively less atrophy than the other brain lobes. Sex, age, and magnetic field strength had greater direct impacts on structural indicators than APOE genotype and education. The analysis of interaction effects revealed that the APOE ε4 mutation carriers exhibited more severe structural changes in specific brain regions as the BAD increased. However, sex, age, and education had minimal regulatory influence on the structural changes associated with BAD.

**Conclusion:**

Longitudinal analysis, with the onset time point of AD as the reference, can accurately describe the features of structural changes preceding the onset of AD and provide a comprehensive understanding of AD development.

## 1 Introduction

Alzheimer’s disease (AD) is a chronic neurodegenerative disease that is the primary cause of dementia and progressively impairs various cognitive functions (Dicks et al., 2020; Ji et al., 2021). AD is characterized by the accumulation of extracellular amyloid-β (Aβ) and intracellular phosphorylated tau protein, as well as neuronal and synaptic loss, along with a reduction in cerebral cortex cells (Hellström-Lindahl et al., 2009; Hyman et al., 2012; Ji et al., 2021). Numerous studies have focused on measuring AD-related biochemical markers, particularly imaging biomarkers. These imaging biomarkers present an opportunity to improve patient diagnosis and investigate the progression of AD pathophysiology (La Joie et al., 2020). Among these imaging biomarkers, atrophy of brain structures serves as a crucial biomarker for AD clinical trials and for estimating disease progression. Previous studies have characterized AD by significant gray matter atrophy in various brain regions, including the medial temporal lobe, parietal lobe, frontal lobe, posterior cingulate cortex, thalamus, and corpus callosum cingulate (Schuff et al., 2009; Dicks et al., 2020; Dong et al., 2021; Wei et al., 2023). Longitudinal trajectory analyses have also demonstrated progressive atrophy in specific brain regions, such as the hippocampus, amygdala, and entorhinal cortex, during the conversion process of AD (Younes et al., 2019).

Previous studies have indicated the impact of age, sex, and APOE genotype on brain atrophy in relation to AD. Aging has been established as a significant risk factor for both AD and brain atrophy (Guerreiro and Bras, 2015). Notably, patients with early-onset AD exhibited more severe and rapid brain atrophy than older patients (Cho et al., 2013a). Furthermore, some research findings demonstrated that women experienced faster brain atrophy than men in mild cognitive impairment (MCI) and AD patients (Beam et al., 2018; Nebel et al., 2018). Additionally, APOE ε4 carriers exhibited a more pronounced loss of gray matter than noncarriers in AD and MCI patients (Taylor et al., 2014; Susanto et al., 2015). Magnetic field strength is an important parameter of magnetic resonance imaging (MRI) which is related to MRI acquisitions. Therefore, the magnetic field strength may affect the results of studies on brain atrophy characteristics by affecting brain measures based on structural MRI. Some previous studies involving MRI used magnetic field strength as control variable (van Loenhoud et al., 2022). Education is a factor that can influence brain atrophy (Arenaza-Urquijo et al., 2013). However, studies on the influence of education on brain atrophy have been rare. Moreover, most studies have focused on individuals or a few factors, with few examining the combined impact of all these factors on AD-related brain atrophy.

Previous studies on AD-related brain atrophy have yielded inconsistent and sometimes contradictory findings. For instance, certain cross-sectional studies have reported cortical thinning in relation to brain amyloidosis (Dickerson et al., 2009; Becker et al., 2011), while others have found no association or even observed increased cortical thickness (Josephs et al., 2008; Chételat et al., 2010). Moreover, the majority of studies have taken a cross-sectional approach, comparing volume, surface area, or cortical thickness differences between AD patients and healthy controls to investigate AD-related brain atrophy. Limited research has focused on longitudinal structural atrophy prior to the onset of AD. Previous longitudinal studies commonly use the subject’s initial observation time point as the baseline and artificially treat subjects in different stages before AD onset as though they have the same starting point. In a study conducted by Gordon et al. (2018), the time to symptom onset was estimated for participants and defined as the participant’s current age minus the age of symptom onset to facilitate a longitudinal analysis of neuroimaging biomarkers in autosomal dominant Alzheimer’s disease. The actual time to AD onset may more accurately reflect the subject’s condition. Therefore, in longitudinal studies which examined brain structural changes preceding AD onset, results obtained from the time to AD onset may be more accurate and effective than those obtained using the subject’s initial observation time point as the baseline.

Our study investigated the atrophic characteristics of brain structures prior to the onset of AD using a longitudinal cohort based on the time to actual AD onset. The time to actual onset of AD was calculated by subtracting the visit time at AD onset from the participant’s visit time during the follow-up period. Thus, our study considered each participant’s actual AD onset time point as the reference point and retrospectively examined their pre-onset state. Additionally, we examined the effects of age, sex, APOE genotype, magnetic field strength, and education on longitudinal brain atrophic characteristics before the onset of AD.

## 2 Materials and methods

### 2.1 Participants

The data utilized in this article were sourced from the Alzheimer’s Disease Neuroimaging Initiative (ADNI) database^1^. ADNI was approved by the institutional review boards of all participating sites (https://adni.loni.usc.edu/wpcontent/uploads/how_to_apply/ADNI_Acknowledgement_List.pdf). Written informed consent was obtained from all individuals. The ADNI, established in 2003, is a collaborative effort involving the National Institute on Aging (NIA), the National Institute of Biomedical Imaging and Bioengineering, the Food and Drug Administration (FDA), and private pharmaceutical companies. We obtained longitudinal clinical and structural imaging data from the ADNI datasets through the Image Data Archive at the Laboratory of Neuroimaging (IDA).^2^

**FIGURE 1:**
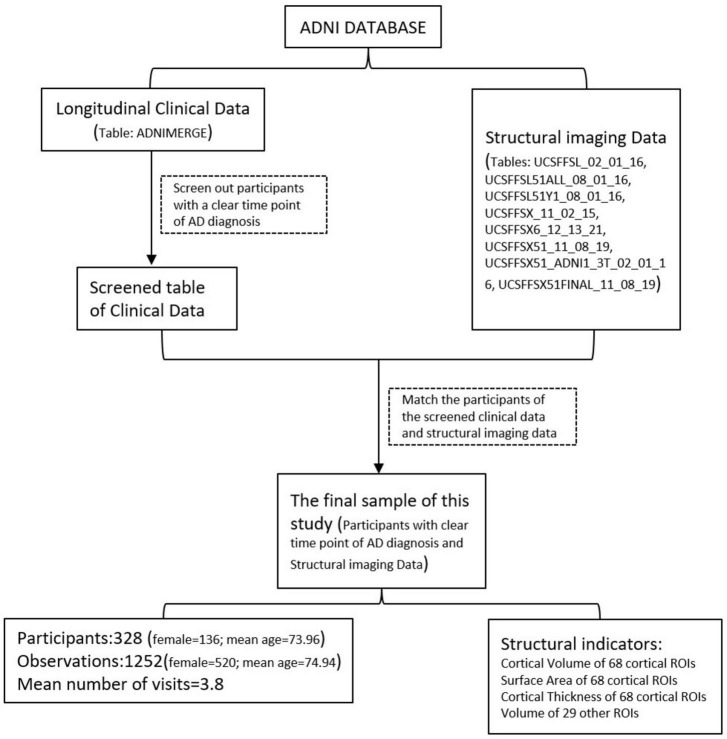
Flowchart of the data screening procedure.

For the participants in the ADNI study, we conducted an analysis of the clinical data available in the ADNI repository (ADNIMERGE table, downloaded on 23 May 2021). We screened participants with a specific time point showing AD onset. This time point was the visit time when the participants were clinically diagnosed with AD within one year of the follow-up interval, and the clinical diagnoses before this visit were mild cognitive impairment or cognitively normal people. To ensure accuracy, we recorded the RIDs (Participant IDs) and the visit time at AD onset for these selected participants. Subsequently, we proceeded to match the RIDs with the tables containing the structural imaging data (UCSFFSL_02_01_16, UCSFFSL51ALL_08_01_16, UCSFFSL51Y1_08_01_16, UCSFFSX_11_02_15, UCSFFSX6_12 _13_21, UCSFFSX51_11_08_19, UCSFFSX51_ADNI1_3T_02 _01_16, UCSFFSX51FINAL_11_08_19 tables, downloaded May 23, 2021). Finally, we included participants in our study who had confirmed time points indicating the onset of AD, along with the corresponding structural imaging data. The flowchart of the data screening process is shown in Figure 1.

In this longitudinal study, we referred to the period before the onset of AD as the BAD (before AD) period. The BAD was calculated by subtracting the visit time at AD onset from the participant’s visit time during follow-up. Therefore, in our study, we determined the pre-onset position of each participant based on their individual onset time point, which served as the reference point.

### 2.2 MR analysis

Detailed information regarding MRI acquisition and preprocessing can be found on the website http://adni.loni.usc.edu/methods/mri-tool/mri-analysis/. The structural MRI brain scans were acquired using both 1.5 T and 3 T MRI scanners. The processing method for structural magnetic resonance followed the UCSF FreeSurfer methods as follows: Cortical reconstruction and volumetric segmentation were performed using the FreeSurfer image analysis suite, which is extensively documented and available for free download online.^3^ Briefly, this processing includes motion correction and averaging of multiple volumetric T1 weighted images, removal of non-brain tissue using a hybrid watershed/surface deformation procedure, automated Talairach transformation, segmentation of the subcortical white matter and deep gray matter volumetric structures, intensity normalization, tessellation of the gray matter white matter boundary, automated topology correction, and surface deformation following intensity gradients to optimally place the gray/white and gray/cerebrospinal fluid borders at the location where the greatest shift in intensity defines the transition to the other tissue class.^4^ The technical procedures used are described in previous publications (Dale and Sereno, 1993; Dale et al., 1999; Fischl and Dale, 2000; Fischl et al., 2001, 2002). The quality control of the FreeSurfer output data was conducted by the UCSF group.

Our study included a total of 97 regions of interest (ROIs). These 97 ROIs consisted of 68 cortical ROIs, 14 subcortical ROIs, 4 ROIs in the ventricular system, 5 ROIs in the corpus callosum, bilateral choroid plexus, cerebrospinal fluid (CSF), hypo-intensity area of white matter, brainstem, and subcortical gray matter (GM). The 68 cortical ROIs included 22 ROIs in the frontal lobe, 10 ROIs in the parietal lobe, 14 ROIs in the temporal lobe, 10 ROIs in the occipital lobe, 10 ROIs in the limbic lobe, and 2 ROIs in the insula. The 14 subcortical ROIs consisted of bilateral hippocampus, bilateral amygdala, bilateral thalamus, bilateral caudate, bilateral putamen, bilateral pallidum, and bilateral nucleus accumbens. A comprehensive list of brain regions can be found in Supplementary materials. The cortical volume (CV), surface area (SA), and average thickness (TA) of 68 cortical ROIs, as well as the volume of other 29 ROIs, were obtained from structural MR images using the UCSF FreeSurfer Method. The volume of each ROI was corrected for total intracranial volume.

### 2.3 Statistical analysis

The statistical analysis was conducted using Stata (version 16.0; Stata Corp.). We utilized multivariate linear mixed effects regression models (LMER) with unbalanced panel data to investigate longitudinal changes in brain structure prior to the onset of AD. The LMER includes both fixed and random effects in the modeling of a response variable. Fixed effects are the variables that are of interest to our study and random effects are associated with the variability of each subject in the study. These models provide flexibility in handling an uneven number of measurement points or intervals.

Within the models, the dependent variables consisted of structural indicators (y_*ij*_: CV, SA, TA, and SV), while the independent variable was the time preceding AD onset (referred to as BAD). Control variables included age, sex, APOE status, magnetic field strength (abbreviated as MFS), the participant’s clinical diagnosis upon enrollment in ADNI (abbreviated as PCD), and years of education. Furthermore, we analyzed the interaction effects of age, sex, APOE status, and years of education on BAD. The statistical analysis equations are expressed as follows:


$$\begin{array}{c} {\text{y}}_{ij}\;={\text{ β}}_{1}{\text{BAD}}_{ij}+{\text{β}}_{2}{\text{age}}_{ij}+{\text{β}}_{3}{\text{sex}}_{ij}+{\text{β}}_{4}{\text{APOE}}_{ij}+{\text{β}}_{5}{\text{PCD}}_{ij}+{\text{β}}_{6}\\ \;{\text{MFS}}_{ij}+{\text{β}}_{7}{\text{education}}_{ij}+{\text{β}}_{8}{\text{BAD\_age}}_{ij}{\text{+β}}_{9}{\text{BAD\_sex}}_{ij}+\\ {\text{β}}_{9}{\text{BAD\_APOE}}_{ij}+{\text{β}}_{12}{\text{BAD\_education}}_{ij}+u_{0\text{j}}+e_{0\text{j}} \end{array}$$

In the linear mixed regression analysis, we determined the within coefficient βn to illustrate the relationship between BAD and the brain structural indicators. Thus the coefficients of independent variables represent the within-cluster correlation, which can be calculated in the LMER model using a fixed effects estimator. Sex was coded as 0 for women and 1 for men. APOE was assigned a value of 0 for noncarriers and 1 for ε4 carriers. MFS was coded as 0 for 1.5T and 1 for 3T. PCD had a value of 0 for cognitively normal individuals (CNs) and 1 for mild cognitive impairment (MCI). In this study, the *p*-values of regression coefficients were adjusted according to the method of Benjamini/Hochberg (B/H) to control the false discovery rate (FDR). A regression coefficient was considered to be statistically significant, if its corresponding B/H-adjusted *p*-value was below 0.05.

## 3 Results

### 3.1 Demographics

After carefully reviewing clinical tables and tables of structural imaging data, we included 328 participants who exhibited specific time points indicating the onset of AD and provided corresponding structural imaging data. The average number of follow-up visits was 3.8, with an interval of 6 months between each visit. Within this cohort, there were 136 females and 192 males. Upon enrollment in the ADNI, 26 individuals were diagnosed as cognitively normal, while 302 were diagnosed with mild cognitive impairment. Furthermore, 120 participants were APOE ε4 carriers, while 206 participants were noncarriers. The average number of years of education in this cohort was 15.93. The observational dataset comprised a total sample of 1,252 patients. Among these observational samples, the mean age was 74.94 years, ranging from 55 to 91 years. A total of 710 samples were scanned using a 1.5T machine, while 542 samples were scanned using a 3T machine. The average BAD was −28.15 months, ranging from −156 to 0 months. The detailed demographic information can be found in Table 1.

**TABLE 1:** Demographics of the study sample.

Descriptive measures	| BAD| (month)				
	0	0–12	13–24	25–60	> 60
Number of subjects	328	261	164	162	47
Age (years) M ± SD (range)	75.30 ± 7.14 (56–91)	74.70 ± 7.14 (56–91)	74.45 ± 7.22 (56–89)	75.33 ± 6.47 (55–88)	75.84 ± 6.31 (60–88)
Sex (female/male)	136/192	110/151	68/96	71/91	16/31
Field (1.5T/3T)	195/133	141/120	92/72	90/72	36/11
APOE (noncarriers/carriers)*	120/206	79/182	92/72	67/95	27/20
PCD (CN/MCI)	26/302	26/302	26/302	26/302	26/302
Education (years) M ± SD (range)	15.93 ± 2.76 (6–20)	15.83 ± 2.81 (6–20)	16.02 ± 2.81 (8–20)	16.01 ± 2.67 (8–20)	15.86 ± 2.53 (10–20)
MMSE M ± SD (range)	24.04 ± 2.87 (15–30)	25.29 ± 2.73 (19–30)	26.59 ± 1.99 (19–30)	27.39 ± 2.03 (19–30)	28.85 ± 1.4 (23–30)
CDRSB M ± SD (range)	4.22 ± 1.45 (1–10)	3.13 ± 1.53 (1–10)	2.14 ± 1.15 (0–5.5)	1.68 ± 1.05 (0–5)	0.69 ± 0.69 (0–2.5)
ADAS11 M ± SD (range)	17.23 ± 6.29 (7–56)	15.49 ± 5.81 (3–56)	12.86 ± 4.35 (0–31)	10.64 ± 4.01 (0–23)	7.52 ± 3.67 (1–17)
ADAS13 M ± SD (range)	26.89 ± 7.79 (9–71)	24.47 ± 7.34 (5–71)	20.71 ± 6.03 (0–43)	17.44 ± 6.03 (1–33)	12.52 ± 5.84 (2–28)
ADASQ4 M ± SD (range)	8.31 ± 1.79 (1–10)	7.78 ± 1.99 (2–10)	6.97 ± 2.14 (0–10)	6.05 ± 2.47 (0–10)	4.33 ± 2.59 (0–10)

PCD, participant’s clinical diagnosis when first enrolled in the ADNI; CN, cognitively normal people; MCI, mild cognitive impairment; MMSE, mini-mental state examination; ADAS11, 11-item Alzheimer’s disease assessment scale-cognitive subscale (ADAS-cog 11); ADAS13, 13-item Alzheimer’s disease assessment-cognitive subscale (ADAS-cog 13); CDR, clinical dementia rating; CDRSB, the CDR sum of boxes. | BAD| represents the absolute value of BAD. *APOE ε4 carriers indicate subjects with one or two copies of the ε4 allele. Continuous variables are shown as the mean ± standard deviation.

### 3.2 Changes in brain structure before the onset of AD

The detailed results of the multivariate linear mixed-effects regression in the 97 ROIs were presented in the Supplementary materials. After controlling for the effects of age, sex, APOE status, magnetic field strength, participants’ clinical diagnosis and years of education, we found that the cortical volume of 16 cortical ROIs (4 ROIs in frontal lobe, 1 ROI in parietal lobe, 5 ROIs in temporal lobe, 3 ROIs in occipital lobe, 3 ROIs in limbic lobe), the surface area of 14 cortical ROIs (5 ROIs in frontal lobe, 1 ROI in parietal lobe, 2 ROIs in temporal lobe, 4 ROIs in occipital lobe, 2 ROIs in limbic lobe) and the cortical thickness of 15 cortical ROIs (2 ROIs in frontal lobe, 8 ROIs in temporal lobe, 1 ROI in occipital lobe, 3 ROIs in limbic lobe, 1 ROI in insula) gradually atrophied with decreasing distance to the onset of AD. These brain regions were mainly distributed in the frontal lobe and temporal lobe, and that atrophy in the parietal and occipital lobes was less common than that in other brain lobes.

**FIGURE 2:**
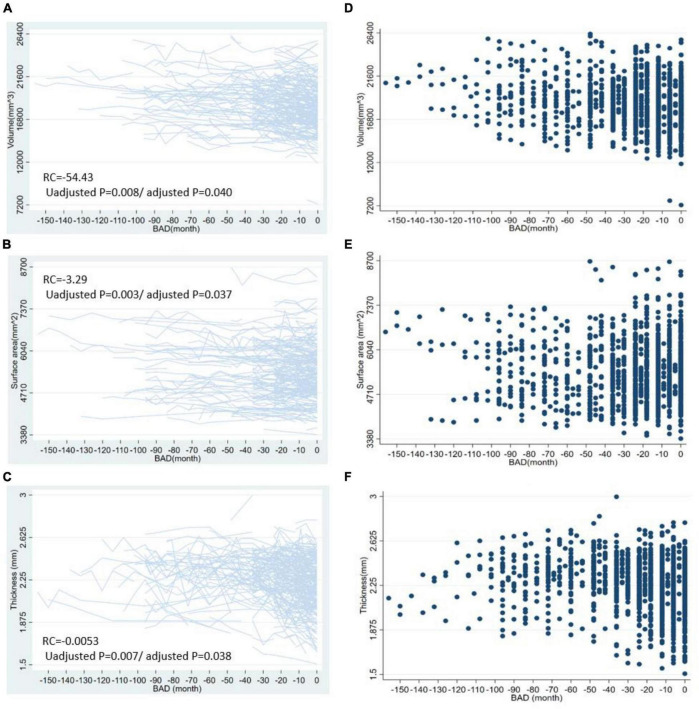
The longitudinal distributions and scatter plots of some cortical indicators. This figure shows the longitudinal distribution of cortical volume in left superior frontal lobe **(A)**, surface area in left rostral middle frontal lobe **(B)**, the cortical thickness in left caudal middle frontal lobe **(C)** and the scatter plots of cortical volume in left superior frontal lobe **(D)**, surface area in left rostral middle frontal lobe **(E)**, and left caudal middle frontal lobe **(F)**. BAD, the time before the onset of Alzheimer’s disease. RC, regression coefficients; the regression coefficients listed in the figure are the nonstandard regression coefficients.

**FIGURE 3:**
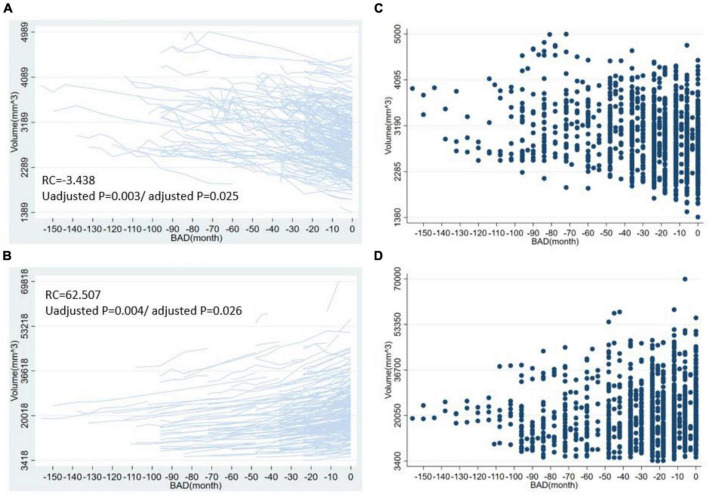
The longitudinal distribution and scatter plots of volume in right hippocampus and right lateral ventricle. This figure shows the longitudinal distribution of volume in the right hippocampus **(A)**, right lateral ventricle **(B)** and the scatter plots of volume in the right hippocampus **(C)**, right lateral ventricle **(D)**. BAD, the time before the onset of Alzheimer’s disease. RC, regression coefficients; the regression coefficients listed in the figure are the nonstandard regression coefficients.

For the volume of cortical regions, the volume of the left superior frontal lobe exhibited the greatest change with BAD (RC = −54.43 [−94.33, −14.53], *p* = 0.008, adjusted *p* = 0.0397). For the surface area of the cortical regions, the surface area of the left rostral middle frontal lobe showed the greatest change with BAD (−3.29 [−5.49, −1.09], *p* = 0.003, adjusted *p* = 0.0368). For the thickness of the cortical regions, the cortical thickness of the left caudal middle frontal lobe showed the greatest change with BAD (−0.0053 [−0.0092, −0.0015], *p* = 0.007, adjusted *p* = 0.0379). The statistically significant regression coefficients of BAD were listed in Table 2. The longitudinal distributions and scatter plots of cortical volume in left superior frontal lobe, surface area in left rostral middle frontal lobe, and cortical thickness in left caudal middle frontal lobe were shown in Figure 2. We found that the volumes of the subcortical GM (−1775.34 [−2992.84, −557.83], *p* = 0.004, adjusted *p* = 0.027), right hippocampus, left amygdala, right putamen, right choroid plexus, corpus callosum and brainstem gradually decreased with decreasing distance to the onset of AD. The volumes of the right lateral ventricle increased before the onset of AD, which also demonstrated atrophy of the subcortical brain parenchyma. The regression coefficients of BAD in above brain regions were listed in Table 2. The longitudinal distributions and scatter plots of the volume in the right hippocampus and right lateral ventricle were shown in Figure 3.

**TABLE 2:** The regression coefficients for BAD.

ROI	RC (*P*/adjusted *P*)	ROI	RC (*P*/adjusted *P*)	ROI	RC (*P*/adjusted *P*)	ROI	RC (*P*/adjusted *P*)	ROI	RC (*P*/adjusted *P*)
Superior frontal (R)SA	−3.145 (0.001/0.019)	Caudal middle frontal (L)SA	−1.185 (0.002/0.033)	Caudal middle frontal (L)TA	−0.005 (0.007/0.038)	Pars orbitalis (L)CV	−2.161 (0.003/0.035)	Pars orbitalis (L)SA	−0.936 (< 0.001/< 0.0233)
Precentral gyrus (L)SA	−1.529 (0.005/0.031)	Rostral middle frontal (L)SA	−3.289 (0.003/0.037)	Rostral middle frontal (L)TA	−0.005 (0.008/0.039)	Superior frontal (L)CV	−54.431 (0.008/0.040)	Caudal middle frontal (R)CV	−4.212 (0.002/0.031)
Lateral orbital frontal (R)CV	−4.690 (0.004/0.031)	Superior parietal (L)CV	−31.372 (0.003/0.033)	Inferior parietal (R)SA	−2.525 (0.010/0.046)	Superior temporal (R)CV	−6.960 (0.007/0.037)	Superior temporal (R)TA	−0.002 (< 0.001/< 0.0233)
Temporal pole (R)CV	−3.159 (0.007/0.036)	Temporal pole (R)TA	−0.004 (0.003/0.030)	Transverse temporal (R)TA	−0.0016 (0.001/0.018)	Middle temporal Gyrus (L)SA	−3.206 (< 0.001/< 0.0233)	Middle temporal gyrus (L)TA	−0.002 (< 0.001/ < 0.0233)
Temporal Pole (L)CV	−3.413 (0.004/0.03)	Temporal pole (L)TA	−0.003 (0.005/0.029)	Entorhinal cortex (R)CV	−3.541 (0.003/0.032)	Entorhinal cortex (R)TA	−0.005 (0.002/0.029)	Inferior temporal (R)CV	−9.042 (0.007/0.035)
Inferior temporal (R)SA	−2.378 (0.008/0.038)	Middle temporal gyrus (R)TA	−0.0019 (0.005/0.031)	Banks superior temporal sulcus (R)TA	−0.0017 (0.005/0.030)	Cuneus (L)CV	−1.835 (0.004/0.029)	Lingual gyrus (R)CV	−4.832 (0.003/0.028)
Cuneus (L)TA	−0.004 (0.009/0.042)	Fusiform gyrus (L)SA	−3.080 (< 0.001/< 0.0233)	Lateral occipital (R)SA	−2.533 (0.01/0.045)	Fusiform gyrus (R)CV	−8.219 (0.005/0.028)	Fusiform gyrus (R)SA	−2.445 (0.003/0.029)
Lingual gyrus (R)SA	−2.092 (0.003/0.027)	Posterior cingulate (R)TA	−0.0014 (< 0.001/< 0.0233)	Rostral anterior cingulate (R)CV	−2.608 (0.003/0.026)	Caudal anterior cingulate (L)TA	−0.00198 (< 0.001/< 0.0233)	Posterior cingulate (L)CV	−1.74517 (0.002/0.027)
Posterior cingulate (L)SA	−1.05393 (< 0.001 < 0.0233)	Posterior cingulate (L)TA	−0.001 (0.011/0.047)	Posterior cingulate (R)CV	−2.26589 (0.01/0.044)	Rostral anterior cingulate (R)SA	−0.962 (0.002/0.026)	Insula (L)TA	−0.00103 (0.004/0.028)
Putamen (R)SV	−15.3559 (0.004/0.027)	Amygdala (L)SV	−0.99765 (0.006/0.033)	Hippocampus (R)SV	−3.4376 (0.003/0.025)	Central corpus callosum (SV)	−2.84388 (< 0.001/< 0.0233)	Middle posterior corpus callosum (SV)	−3.46545 (< 0.001/< 0.0233)
Choroid plexus (R)SV	−4.54889 (< 0.001/< 0.0233)	Brain stem (SV)	−6.38759 (0.003/0.024)	Lateral ventricle (R)SV	62.50721 (0.004/0.026)	Subcortical GM	−1775.337 (0.004/0.027)		

The regression coefficients listed in the table were the non-standard regression coefficients. The unadjusted and adjusted *P*-values according to the Benjamini/Hochberg (B/H) were listed in brackets. In this table, only the regression coefficients with statistical significance after adjusted were listed. For other detailed regression coefficients, please refer to the supplementary electronic tables. RC, regression coefficients; (L), left lateral; (R), right lateral; CV, cortical volume, unit: mm^3^; SA, surface area, unit: mm^2^; TA, average thickness, unit: mm. BAD, time before the diagnosis of Alzheimer’s disease, unit: month. SV = subcortical volume, unit: mm^3^.

### 3.3 Effects of age, sex, APOE, magnetic field strength and education on brain structure

#### 3.3.1 Effect of age on brain structure

The regression coefficients for age in 97 ROIs were listed in Supplementary materials. The cortical volume in 60 ROIs (19 ROIs in frontal lobe, 8 ROIs in parietal lobe, 13 ROIs in temporal lobe, 10 ROIs in occipital lobe, 8 ROIs in limbic lobe, 2 in insula), cortical thickness in 57 ROIs (21 ROIs in frontal lobe, 9 ROIs in parietal lobe, 13 ROIs in temporal lobe, 9 ROIs in occipital lobe, 3 ROIs in limbic lobe, 2 ROIs in insula) and cortical surface area in 20 ROIs (3 ROIs in frontal lobe, 3 ROIs in parietal lobe, 3 ROIs in temporal lobe, 5 ROIs in occipital lobe, 6 ROIs in limbic lobe) atrophied with age. For the volume of cortical regions, the volume of the left superior frontal lobe exhibited the greatest change with age (−69.48 [−105.72, −33.24], *p* < 0.0001, adjusted *p* < 0.0233). For the surface area of the cortical regions, the surface area of the left lateral occipital lobe showed the greatest change with age (−23.18 [−38.81, −7.55], *p* = 0.004, adjusted *p* = 0.008). For the thickness of the cortical regions, the cortical thickness of the left entorhinal cortex showed the greatest change with age (−0.0218 [−0.0293, −0.0143], *p* < 0.0001, adjusted *p* < 0.0233).

The age-related atrophy involved greater regions for cortical thickness and cortical volume than cortical surface area. The age-related atrophy was more extensive than AD-related atrophy. In addition, the atrophy caused by AD was mainly distributed in the frontal and temporal lobes and less common in the parietal and occipital lobes, while the atrophy caused by aging involved all the brain. The cortical volume of 27 ROIs, the surface area of 37 ROIs and the cortical thickness of 33 ROIs were atrophied with aging and not atrophied with BAD. These brain regions were listed in Table 3.

**TABLE 3:** Summary of the regions of cortical structure atrophied with age and not with BAD.

	Parameter	Brain regions#
Frontal lobe	CV	8 [Pars orbitalis (R), pars triangularis (L), superior frontal (R), pars opercularis (L), precentral gyrus (R), rostral middle frontal (L), rostral middle frontal (R), caudal middle frontal (L)]
	SA	9 [Pars triangularis (L), paracentral gyrus (L), superior frontal (L), rostral middle frontal (R), pars opercularis (L), precentral gyrus (R), lateral orbital frontal (L), caudal middle frontal (R), medial orbital frontal (L)]
	TA	11 [Pars triangularis (L), superior frontal (L), lateral orbital frontal (L), medial orbital frontal (L), lateral orbital frontal (R), precentral gyrus (R), superior frontal (R), precentral gyrus (L), pars triangularis (R), rostral middle frontal (R), pars orbitalis (R)]
Parietal lobe	CV	6 [Supramarginal gyrus (L), superior parietal (R), postcentral gyrus (L), precuneus (R), inferior parietal (R), precuneus (L)]
	SA	8 [Supramarginal gyrus (L), precuneus (R), superior parietal (R), superior parietal (L), precuneus (L), supramarginal gyrus (R), postcentral gyrus (R), inferior parietal (L)]
	TA	8 [Superior parietal (L), supramarginal gyrus (R), superior parietal (R), precuneus (R), supramarginal gyrus (L), postcentral gyrus (L), inferior parietal (L), inferior parietal (R)]
Temporal lobe	CV	6 [Middle temporal gyrus (R), middle temporal gyrus (L), banks superior temporal sulcus (L), entorhinal cortex (L), transverse temporal (L), superior temporal (L)]
	SA	9 [Superior temporal (L), transverse temporal (L), banks superior temporal sulcus (L), banks superior temporal sulcus (R), middle temporal gyrus (R), superior temporal (R), inferior temporal (L), transverse temporal (R), entorhinal cortex (L)]
	TA	4 [Banks superior temporal sulcus (L), inferior temporal (R), entorhinal cortex (L), inferior temporal (L)]
Occipital lobe	CV	3 [Lateral occipital (R), fusiform gyrus (L), lingual gyrus (L)]
	SA	3 [Cuneus (L), lateral occipital (L), pericalcarine (R)]
	TA	6 [Fusiform gyrus (R), lateral occipital (R), cuneus (R), lingual gyrus (R), lingual gyrus (L), lateral occipital (L)]
Limbic lobe	CV	2 [Parahippocampus (L), parahippocampus (R)]
	SA	6 [Caudal anterior cingulate (R), rostral anterior cingulate (L), posterior cingulate (R), parahippocampus (L), isthmus cingulate (R), isthmus cingulate (L)]
	TA	3 [Isthmus cingulate (L), parahippocampus (L), caudal anterior cingulate (R)]
Insula	CV	2 [Insula (L), insula (R)]
	SA	2 [Insula (L), insula (R)]
	TA	1 [Insula (R)]

#Data are the number of brain regions. “(L)” indicates the left lateral. “(R)” indicates the right lateral. CV, cortical volume, unit: mm^3^; SA, surface area, unit: mm^2^; TA, thickness average, unit: mm. BAD, time before the diagnosis of Alzheimer’s disease, unit: month. AD, Alzheimer’s disease.

#### 3.3.2 Effects of sex on brain structure

The regression coefficients for sex in 97 ROIs were listed in Supplementary materials. The regression coefficients of sex were positively statistically significant for the cortical volume in 65 ROIs and cortical surface area in 66 ROIs. In our study, the value of sex was 0 for women and 1 for men. Therefore, males had greater cortical volume and cortical surface area in almost the whole-brain cortical regions. However, the regression coefficients of sex were negatively statistically significant for the cortical thickness in 13 ROIs (9 in frontal lobe, 4 in limbic lobe). Therefore, in these regions, males had thinner cortical thickness compared to females. In addition, males had greater volume than females in the corpus callosum, bilateral choroid plexus, CSF, brainstem, subcortical GM and the ventricular system except the fourth ventricle.

#### 3.3.3 Effect of magnetic field strength on brain structure

The regression coefficients of magnetic field strength were positively statistically significant for the cortical volume in 62 ROIs, cortical surface area in 21 ROIs, cortical thickness in 62 ROIs. In our study, the magnetic field strength was 0 for 1.5T and 1 for 3T. Therefore, the cortical volume, surface area and cortical thickness in these regions were greater with the 3T scanner than with the 1.5T scanner. However, the surface area of 22 ROIs and the subcortical volume of 3 ROIs were greater with the 1.5T scanner than with the 3T scanner. Thus, the values of structural indicators of the brain, especially volume and cortical thickness, were affected by the magnetic field strength of the MRI scanner.

#### 3.3.4 Effects of APOE and education on brain structure

The regression coefficients of APOE status were negatively statistically significant for the cortical volume in 2 ROIs, cortical surface area in 3 ROIs, cortical thickness in 2 ROIs, and the volume in left amygdala. In our study, the value of APOE status was 0 for noncarriers and 1 for carriers. Therefore, the structural indices of these regions were smaller in participants carrying the mutant gene than in noncarriers. In addition, the cortical volume in left caudal middle frontal lobe and cortical thickness in 4 ROIs decreased with the increase of years of education. While the cortical surface area and cortical thickness of right pars triangularis increased with the increase of years of education. Therefore, the structural indices of fewer brain regions were affected by APOE and education than age, sex and magnetic field strength.

#### 3.4. Interaction effects of sex, age, APOE, and education on brain structural changes in patients with BAD

The regression coefficients of the interaction term between BAD and age were positively statistically significant for the cortical volume in 2 ROIs (left superior parietal lobe and right entorhinal cortex), cortical thickness in left caudal anterior cingulate, and volume in right putamen, central corpus callosum, middle posterior corpus callosum, and subcortical GM. Therefore, the structural indicators were more severely atrophied in participants who were younger in these regions.

The regression coefficients of the interaction term between BAD and sex were positively statistically significant for the cortical volume in 4 ROIs (left cuneus, left posterior cingulate, right caudal middle frontal lobe and right lateral orbital frontal lobe), and for cortical thickness in 8 ROIs. Therefore, the structural indicators were more severely atrophied in participants who were female in these regions.

The regression coefficients of the interaction term between BAD and APOE were negatively statistically significant for the cortical volume in 2 ROIs (right caudal middle frontal lobe and left superior parietal lobe), cortical surface area in 1 ROI (left caudal middle frontal lobe), cortical thickness in 4 ROIs and volume in right choroid plexus. Therefore, the structural indicators showed more severe atrophy in participants carrying the APOE mutant gene in these regions.

The regression coefficient of the interaction term between BAD and education was negatively statistically significant for the cortical surface area in right transverse temporal lobe and left temporal pole. Therefore, these structural indicators were more severely atrophied in participants who had more years of education.

## 4 Discussion

In our study, we examined the atrophic characteristics of the brain prior to the onset of Alzheimer’s disease (AD) using the time of AD onset as a reference. We also investigated the influence of sex, age, APOE genotype, magnetic field strength, and education on brain structure, as well as their regulatory effects on brain atrophy related to BAD. The results revealed significant atrophy in various brain regions before the onset of AD, particularly in the frontal and temporal lobes. Notably, the parietal and occipital lobes exhibited relatively less atrophy than other brain lobes. Sex, age, and magnetic field strength directly impacted structural indicators in most brain regions. However, the influence of APOE genotype and education on structural indices was limited to specific brain regions. Specifically, male participants, younger individuals, and those scanned with 3T demonstrated larger values for structural indicators than female participants, older individuals, and those scanned with 1.5T. Interestingly, males exhibited thinner cortical thickness than females. Moreover, we observed a different distribution of BAD-related atrophy and age-related atrophy. Finally, APOE ε4 carriers displayed more severe structural changes associated with BAD in some brain regions, while the regulatory impact of sex, age, magnetic field strength, and education on the structural changes related to BAD was minimal.

Previous research has indicated the presence of extensive cortical and subcortical brain atrophy prior to the onset of AD (Mak et al., 2017; Bernstein et al., 2021; Li et al., 2022; Nakazawa et al., 2022). The distribution of brain atrophy before the onset of AD in our study was basically the same as in previous research. Previous studies have highlighted the occurrence of brain atrophy in specific regions before the onset of AD, including some cortical regions in the frontal and temporal lobes, as well as the hippocampus and amygdala in subcortical structures (Apostolova et al., 2015; Mak et al., 2017; Dicks et al., 2019; Kwak et al., 2022; Li et al., 2022; Nakazawa et al., 2022). According to previous research, brain atrophy before the onset of AD may be attributed to neuronal death and synapse loss (Apostolova et al., 2015; Vipin et al., 2018; Moore et al., 2020; Martersteck et al., 2021). In addition, previous studies also have indicated that atrophy of the occipital lobe was relatively less severe than other brain lobes before the onset of AD (Nosheny et al., 2019; Timmers et al., 2019; Zhuo et al., 2021; Gonuguntla et al., 2022). For instance, at the moderate cognitive impairment stage in AD patients, structural abnormalities were observed lesser extent in the occipital lobe than in the temporal lobe and hippocampus (He et al., 2022). Some studies have even reported a greater volume of occipital lobe in patients with MCI or AD than healthy controls (Ryu et al., 2022). Some studies showed that the atrophy of occipital lobe typically did not manifest in the early stages of AD but tended to appear in the later stages (Montembeault et al., 2018). The relatively lighter atrophy of the occipital lobe compared to other brain lobes can be attributed to its role as the region responsible for visual functions. On the other hand, the frontal, temporal, and limbic lobes primarily control functions such as cognition, memory, emotion, and behavior. Therefore, the characteristics of brain atrophy before the onset of AD in this study are basically consistent with previous research results. Extensive brain atrophy prior to AD predominantly affects the frontal lobe, temporal lobe, hippocampus, and amygdala, particularly the frontal and temporal lobes, with less involvement of the occipital lobe. This pattern of atrophy may serve as an important differentiating factor between AD and other neurodegenerative diseases, as well as between AD and normal aging.

Previous studies on the characteristics of brain atrophy before the onset of AD have focused primarily on changes in volume, with limited attention given to cortical surface area and cortical thickness. Our study revealed extensive reductions in both cortical thickness and cortical surface area prior to the onset of AD. Atrophy of cortical thickness and surface area plays a significant role in the progression of AD. For instance, Wu et al. (2021) discovered that a reduced surface area of the temporal pole and decreased thickness of the wedge-shaped gyrus were associated with an increased risk of AD. Another study examined the link between baseline MRI atrophy and cognitive outcomes over a two-year period in early-onset AD patients. The results indicated that baseline hemispheric cortical thickness, as well as regional thickness, predicted subsequent cognitive performance (Contador et al., 2022). Furthermore, a Mendelian randomization analysis suggested that a reduced temporal pole area and parietal cortex thickness were associated with a greater risk of AD (Wu et al., 2021).

In our study, we found direct effects of sex, age, and magnetic field strength on structural indices in most brain regions prior to the onset of Alzheimer’s disease (AD). However, sex and age exerted regulatory influences on BAD-related brain atrophy only in a small number of brain regions, mainly manifested as greater atrophy of women and young individuals than men and elderly individuals in these regions. Previous studies also reported more severe atrophy and cognitive decline in women than in men (Mattsson et al., 2018). Additionally, early-onset AD patients demonstrated faster volume loss and cortical thinning in the medial temporal lobe and specific subcortical brain regions than late-onset AD patients (Cho et al., 2013a,b; Fiford et al., 2018; Lee et al., 2020). Moreover, our study showed that the APOE genotype had a minimal direct impact on brain structure indices. However, the APOE genotype did have a regulatory effect on the rate of brain atrophy in some brain regions before the onset of AD, mainly manifested as APOE ε4 carriers exhibited higher atrophy rates than APOE ε4 noncarriers. Previous studies have also showed that APOE ε4 allele carriers displayed greater rates of brain atrophy and faster cognitive function decline than noncarriers (Shen et al., 2019; Yan et al., 2020; Duarte-Guterman et al., 2021). In addition, the APOE regulation occurs prior to AD conversion, accelerating cerebral atrophy and AD progression (Wei et al., 2023). Potential mechanisms explaining the faster atrophy in certain brain regions among women and APOE carriers have been suggested based on recent metabolic and transcriptomic data (Liu et al., 2013; Hobel et al., 2019; Arnold et al., 2020; Paranjpe et al., 2021). Compared with male and APOE ε4 noncarriers, female and APOE ε4 carriers exhibit greater impairment of mitochondrial energy production (Arnold et al., 2020). Furthermore, *in vitro* and transcriptomic data have indicated that sex modulated neuroinflammation, another risk factor for AD, with stronger inflammatory dysregulation in females with AD (Hanamsagar and Bilbo, 2016; Paranjpe et al., 2021). APOE ε4 also triggered neuroinflammatory cascades, leading to neurovascular dysfunction and leakage of blood-derived toxic proteins into the brain (Bell et al., 2012). These findings suggested a potential model of AD pathogenesis in which the female and the APOE ε4 allele contribute to neuroinflammatory damage to the brain and brain microvasculature, resulting in increased tau and brain atrophy (Iaccarino et al., 2017; Sone et al., 2017). The mechanism underlying the faster atrophy observed before the onset of AD in young men than in older men has yet to be fully elucidated. Some studies suggest that the cognitive reserve theory may account for the faster atrophy observed in early-onset AD patients than in late-onset AD patients (Sone et al., 2017). The distribution of atrophied brain regions regulated by age, sex, and APOE in our study broadly aligns with the findings of previous research. However, compared with previous studies, we observed fewer brain regions regulated by age and sex, as well as a limited number of subcortical brain regions regulated by APOE. This discrepancy may be attributed to the fact that most previous studies on regulatory effects included samples diagnosed with AD, while all samples in our study were collected prior to the onset of AD, potentially resulting in variations in the degree of brain atrophy.

Age directly influenced brain structure prior to the onset of AD, leading to atrophy in a wide range of brain structures. However, the distributions of brain atrophy related to aging and BAD were different. It is manifested as the aging-related atrophy involves all areas of the brain, while atrophy resulting from AD typically affects the frontal and parietal lobes. Hence, by considering the atrophic extent of occipital lobe and parietal lobe, it may be possible to differentiate between individuals at normal aging and those in the pre-onset stage of AD. However, further validation through longitudinal comparative analysis of normal aging and AD cohorts is necessary to confirm the above results. In this study, we observed a minimal direct impact of education duration on brain structure and a minimal regulatory effect on the rate of brain atrophy. Certain brain regions exhibited lower cortical thickness with more years of education, and there was a correlation between more education and a faster rate of brain atrophy. Limited previous research has explored the influence of education on brain atrophy. It has been suggested that educational attainment does not provide protection against cognitive decline caused by brain pathology (Bauer et al., 2020). In fact, one study even indicated that higher education levels were associated with accelerated cognitive decline in patients with dementia (Scarmeas et al., 2006). A recent study showed that the volume of ventricle increased with higher education levels (Chen et al., 2024). Since the increase of ventricle volume indirectly reflected the atrophy of brain parenchyma. Therefore, the higher education could be harmful to some brain structural volume. These findings potentially support our conclusion that higher education levels in specific brain regions are linked to a faster rate of brain atrophy.

Furthermore, our study identified a direct effect of magnetic field strength on the brain structure index, revealing that higher magnetic field strength corresponds to greater brain structure index values. However, previous studies have reported contradictory findings, suggesting that increased magnetic field strength may result in a reduction in brain volume (Marchewka et al., 2014). It is important to note that the previous study included AD patients and utilized SPM software for analysis, whereas our study focused on participants before the onset of AD and utilized FreeSurfer software. Therefore, further investigation is required to determine the positive or negative influence of magnetic field strength on brain structure. Nevertheless, magnetic field strength should be considered a significant control variable when studying the structural characteristics of the brain.

This study has several limitations. First, this study included only the data in the ADNI database and the sample size was relatively small. Whether the data in other databases can reveal similar results needs to be further verified. Future analyses should be conducted in multiple institutional cohorts beyond the ADNI with larger sample sizes to validate this preliminary work. In addition, the number of longitudinal follow-up points before the onset of AD included in this study was relatively small, with an average follow-up time of 3.8. However, further analysis of more longitudinal follow-up time points before the onset of AD is needed to obtain more accurate results. Finally, the control variables in this study only included age, sex, APOE genotype, magnetic field strength and years of education, and additional control variables need to be included in the follow-up study to more accurately describe AD-related brain atrophy.

To conclude, this study investigated the atrophic characteristics of brain structures prior to the onset of AD, using the onset of AD as a reference. The findings indicate that extensive brain regions undergo structural atrophy before the onset of AD, particularly in the frontal, temporal and limbic lobes and the occipital lobe exhibits relatively less atrophy than other brain lobes. Additionally, participants who were APOE ε4 carriers exhibited accelerated atrophy in specific brain regions associated with BAD. Furthermore, this study revealed that sex, age, and magnetic field strength have an impact on the values of brain structures. Consequently, it is crucial to include these variables as control variables for accurately characterizing brain structure. Finally, our findings demonstrated the existence of mismatched brain regions between BAD-related atrophy and age-related atrophy. These mismatched regions may play a critical role in distinguishing between aging and AD, as well as in elucidating the pathogenesis and identifying therapeutic targets for AD.

## Data availability statement

The original contributions presented in this study are included in this article/Supplementary material, further inquiries can be directed to the corresponding author.

## Ethics statement

The studies involving humans were approved by the institutional review boards of all participating sites (https://adni.loni.usc.edu/wpcontent/uploads/how_to_apply/ADNI_Acknowledgement_List.pdf). The participants provided their written informed consent to participate in this study. Written informed consent was obtained from the individual(s) for the publication of any potentially identifiable images or data included in this article.

## Author contributions

YH: Conceptualization, Data curation, Formal analysis, Investigation, Methodology, Project administration, Resources, Software, Validation, Visualization, Writing – original draft, Writing – review & editing. TZ: Data curation, Formal analysis, Investigation, Methodology, Resources, Software, Visualization, Writing – review & editing. WZ: Conceptualization, Funding acquisition, Investigation, Methodology, Project administration, Resources, Supervision, Validation, Visualization, Writing – review & editing.

## Footnotes

1
http://adni.loni.usc.edu/

2
https://ida.loni.usc.edu/login.jsp

3
http://surfer.nmr.mgh.harvard.edu/

4
http://adni.loni.usc.edu/methods/mri-tool/mri-analysis/

## Supplementary material

Supplementary Table 1 The Supplementary materials listed the detailed results of the multivariate linear mixed effects regression analysis. The *P*-values corrected by Benjamini/Hochberg (B/H) were also listed in the Supplementary materials. The regression coefficients listed in the Supplementary materials were the non-standard regression coefficients. Abbreviation: RC, regression coefficients; (L), left lateral; (R), right lateral; CV, cortical volume, unit: mm^3^; SA, surface area, unit: mm^2^; TA, average thickness, unit: mm. BAD, time before the diagnosis of Alzheimer’s disease, unit: month. PCD, the participant’s clinical diagnosis upon enrollment in ADNI; MFS, magnetic field strength.
